# Mollaret meningitis: a case report and literature review

**DOI:** 10.3389/fmed.2025.1719046

**Published:** 2025-12-04

**Authors:** Yi-da Wang, Zun-jing Liu, Cheng-yue Sun, Huai-lian Guo, Hong Jiang

**Affiliations:** Department of Neurology, Peking University People’s Hospital, Beijing, China

**Keywords:** Mollaret meningitis, benign recurrent lymphocytic meningitis, herpes simplex virus-2, inflammation, case report

## Abstract

Mollaret meningitis is a rare benign recurrent aseptic meningitis that manifested as headache and abnormal cerebrospinal fluid. The symptoms are self-limiting, typically resolving within days without sequelae, and the prognosis is favorable. We report a typical case of a 43-year-old patient diagnosed with Mollaret meningitis. The patient’s symptoms eased quickly, but post-symptom remission follow-up revealed that abnormal cerebral spinal fluid pressure and cell counts persisted for over 1 month. This suggests that there may be a long-term inflammatory response associated with Mollaret meningitis.

## 1 Introduction

Mollaret meningitis was first described by Mollaret (1). It manifests as headache and lymphocytic meningitis predominantly. Herpes simplex virus-2 can be identified in cerebrospinal fluid of most patients with Mollaret meningitis. The symptoms typically resolve within days without sequelae. Current literature on its self-limited nature, however, focuses solely on clinical symptom resolution. We conducted a long-term follow-up of the patient and reported in detail the results of the cerebrospinal fluid examinations during the follow-up period.

## 2 Case report

A 43-year-old Chinese male presented with headache, nausea, vomiting, and fever lasting for 6 days. Symptoms such as headache and fever were temporarily relieved after taking ibuprofen but recurred within hours. The patient had a history of viral meningitis 15 years ago with similar symptoms and no sequelae. The patient recalled that his headache 15 years ago resolved quickly after treatment of antiviral drugs, but he remained hospitalized for almost 3 months because of persistently elevated intracranial pressure (Table 1). Physical examination revealed neck stiffness and positive Kernig’s sign, with no rash observed. Both computed tomography (CT) and magnetic resonance imaging (MRI) showed no abnormalities. Cerebrospinal fluid (CSF) was colorless and clear, with prominently elevated pressure (> 350 mmH_2_O). CSF analysis indicated lymphocytosis and elevated protein levels (0.77 g/L), while glucose and chloride levels were normal. Cytological examination showed no Mollaret cell or ghost cell. CSF bacterial smears and cultures were negative. Further CSF metagenomic next-generation sequencing (NGS) detected 3,882 sequences of herpes simplex virus-2 (HSV-2). The patient underwent whole exome sequencing during the visit, but no pathogenic gene mutations were reported (Supplementary Table 1).

**TABLE 1:** Cerebrospinal fluid (CSF) examination results in 15 years ago (2010).

After onset	5d	3w	5w	8w
Pressure (mmH_2_O)	>330	180	220	225
White blood cell (/μL)	270	200	50	18
Mononuclear cell (%)	92	80	65	90
Protein (g/L)	0.52	0.4	0.42	0.1

After 1 week of treatment with acyclovir and mannitol, the patient experienced complete resolution of symptoms. Cerebrospinal fluid (CSF) pressure was 310 mmH_2_O, with persistent lymphocytosis in the CSF. NGS testing detected 5 HSV-2 sequences. Following an additional 2 weeks of treatment, CSF pressure decreased to 240 mmH_2_O, and NGS results became negative for HSV-2, prompting discontinuation of the medications. Cytokine profiling of blood and CSF was performed 2 weeks after disease onset. Only serum IL-8 Elevated (26.85 pg/mL), while CSF cytokine levels remained within normal ranges. The patient was ultimately diagnosed with Mollaret meningitis. Follow-up blood tests at 4 and 8 weeks post-onset revealed persistently elevated IL-8 levels, albeit with a declining trend (Supplementary Table 2). At follow-up visits after discharge, the patient exhibited no sequelae. CSF examination results are summarized in Table 2.

**TABLE 2:** Cerebrospinal fluid (CSF) examination results in 2025.

After onset	1w	2w	4w	8w
Pressure (mmH_2_O)	>330	310	240	240
White blood cell (/μL)	110	152	17	0
Mononuclear cell (%)	95	95	96	0
Protein (g/L)	0.77	0.43	0.33	0.41
HSV-2 number in NGS	3882	5	0	0

## 3 Literature review

Mollaret meningitis (MM), also known as benign recurrent aseptic meningitis or benign recurrent lymphocytic meningitis (BRLM), was first described by Mollaret (1) based on three reported cases. The term “BRLM” reflects its core clinical features. Some researchers propose that “BRLM” is a broader term, while “MM” should be reserved for idiopathic recurrent cases (2). However, due to limited studies, the two terms are often treated as describing the same disease entity.

The etiology of BRLM may be associated with HSV-2 infection. In the three cases initially reported by Mollaret (1), no definitive pathogens were identified in the CSF. Advances in polymerase chain reaction (PCR) technology have since revealed a close relationship between HSV-2 and this condition. Tedder et al. (3) collected 13 patients meeting BRLM criteria and detected HSV-2 DNA in 10 cases and HSV-1 DNA in 1 case via CSF PCR. Similar results were reported by Kupila et al. (4), Farazmand et al. (5), with HSV-2 identified in up to 85% of BRLM patients’ CSF. Conversely, approximately 20% of HSV-2 meningitis patients experience recurrence (6, 7), leading to a BRLM diagnosis. Other viruses, such as Epstein-Barr virus (EBV) (8) and human herpes virus-6 (HHV-6) (9), have also been linked to BRLM but are relatively rare. Additionally, BRLM may correlate with epidermoid cysts (10–12), lymphoma (13), and certain autoimmune disorders (14, 15).

The pathogenesis of BRLM remains unclear. It is generally believed that the interaction among viral, host, and environmental factors contributes to disease development (16). After primary infection, HSV-2 establishes latency in the sacral ganglia, and reactivation under various triggers leads to onset of the disease. A familial case reported by Jones and Snyder (17) suggests potential genetic predisposition . Hait et al. (18) identified several autophagy-related gene variants through whole-exome sequencing in 15 patients. Further analysis revealed that these mutations enhance HSV-2 replication and cell death, though the exact role of autophagy in BRLM pathogenesis remains undefined (19).

The term “BRLM” encapsulates the primary clinical features of the disease. Over 80% of patients are female (6, 16). Meningitis symptoms often emerge abruptly, with headaches present in nearly all patients and constituting the primary reason for seeking medical care. More than half of the patients experience photophobia, phonophobia, fever, and other symptoms (4, 6, 20). The symptoms are self-limiting, typically resolving within days without sequelae, and the prognosis is favorable. However, the disease may recur, with recurrence intervals varying widely from weeks to years (20). Some patients may develop herpetic skin lesions during episodes (4).

Cerebrospinal fluid profile is primarily characterized by lymphocytic pleocytosis, with normal or mildly elevated protein levels and normal glucose levels. Routine microbiological examinations, such as smears and cultures, typically yield negative results, but PCR or NGS may detect HSV-2 or, occasionally, other pathogens. In initial report of three cases, Mollaret (1) described endothelial-like cells in the CSF, termed Mollaret cells, which hold diagnostic significance. These cells are morphologically distinct: large in size, with ill-defined borders, abundant cytoplasm, and large irregular nuclei that may exhibit a footprint-like appearance (21). Immunohistochemical staining confirms their monocytic origin (22). Mollaret cells are most prominent within the first 24 h of disease onset, but rapidly degenerate into “ghost cells” (21, 23). Notably, similar cells may also appear in other viral infections, such as HSV-1 (24), varicella zoster virus (VZV) (25), and West Nile virus (26), precluding a diagnosis based solely on cytological findings.

Benign recurrent lymphocytic meningitis is relatively rare, and there is currently no robust research evaluating the safety and efficacy of various treatment regimens. Given the high detection rate of HSV-2, acyclovir is typically administered during acute episodes. Whether antiviral therapy during remission prevents recurrence remains unclear. A randomized controlled trial (RCT) demonstrated that prophylactic valacyclovir in HSV-2-associated meningitis patients did not reduce relapse rates (27). However, long-term acyclovir prophylaxis in a frequently relapsing patient showed reduced recurrence (17), suggesting that RCTs targeting high-frequency relapse cohorts may yield positive results. Case reports indicate that indomethacin may alleviate symptoms and lower interleukin-6 (IL-6) and tumor necrosis factor-α (TNF-α) levels (28, 29). While the self-limiting nature of BRLM obviates the need for treatment studies, therapeutic strategies for patients with frequent recurrences require further research to minimize recurrence.

## 4 Discussion

Benign recurrent lymphocytic meningitis typically follows a self-limited clinical course with favorable prognosis, where symptoms resolve spontaneously within days without sequelae. Current literature on its self-limited nature, however, focuses solely on clinical symptom resolution, with no studies addressing the recovery timeline of CSF abnormalities during or after acute episodes. In this case, both the clinical presentation and CSF abnormalities aligned with BRLM diagnostic criteria. Post-symptom remission follow-up revealed a gradual decline in CSF pressure and cell counts over >1 month, lagging behind clinical recovery. Notably, intracranial pressure remained elevated during convalescence but caused no headaches, suggesting that inflammation, rather than pressure, drives symptoms. To our knowledge, this is the first case report demonstrating that the recovery of intracranial pressure lags behind symptom resolution of BRLM significantly. Investigation into persistent CSF anomalies may further elucidate BRLM’s pathogenesis.

There was another case report documenting elevated cytokine levels in the CSF of patients with BRLM (30). *In vitro* studies also demonstrated increased cytokine levels in patients with recurrent HSV infections, including interleukin (IL)-12, IL-10, and tumor necrosis factor (TNF)-α (31). In our case, cytokine profiling of blood and CSF was performed 2 weeks after disease onset. Elevated serum IL-8 was detected, while CSF cytokine levels remained within normal ranges. Follow-up blood tests at 4 and 8 weeks post-onset revealed persistently elevated IL-8 levels, albeit with a declining trend. Notably, the patient exhibited sustained intracranial hypertension during recovery, which may be attributable to persistent inflammatory responses.

## 5 Conclusion

While BRLM demonstrates rapid symptomatic resolution, persistent inflammatory responses may result in prolonged elevation of CSF pressure and other abnormalities in patients. Further research is required to clarify the dynamics of CSF abnormalities during recovery, which may enhance our understanding of the disease’s pathogenesis.

## Data Availability

The datasets presented in this article are not readily available because of ethical and privacy restrictions. Requests to access the datasets should be directed to the corresponding author.
